# Negative exploration for pyloric stenosis – Is it preventable?

**DOI:** 10.1186/1471-2431-8-37

**Published:** 2008-09-24

**Authors:** Dhanya Mullassery, Sreelakshmi Mallappa, Raheel Shariff, Ross J Craigie, Paul D Losty, Simon E Kenny, David Pilling, Colin T Baillie

**Affiliations:** 1 Department of Paediatric Surgery, The Royal Liverpool Children's Hospital (Alder Hey), Eaton Road Liverpool L12 2AP, UK; 2 Department of Paediatric Radiology, The Royal Liverpool Children's Hospital (Alder Hey), Eaton Road Liverpool L12 2AP, The University of Liverpool, UK

## Abstract

**Background:**

The diagnosis of infantile hypertrophic pyloric stenosis (IHPS), although traditionally clinical, is now increasingly dependent on radiological corroboration. The rate of negative exploration in IHPS has been reported as 4%. The purpose of our study was to look at elements of supportive clinical evidence leading to positive diagnosis, and to review these with respect to misdiagnosed cases undergoing negative exploration.

**Methods:**

All infants undergoing surgical exploration for IHPS between January 2000 and December 2004 were retrospectively analysed with regard to clinical symptoms, examination findings, investigations and operative findings.

**Results:**

During the study period, 343 explorations were performed with a presumptive diagnosis of IHPS. Of these, 205 infants (60%) had a positive test feed, 269 (78%) had a positive ultrasound scan and 175 (55%) were alkalotic (pH ≥7.45 and/or base excess ≥2.5). The positive predictive value for an ultrasound (US) diagnosis was 99.1% for canal length ≥14 mm, and 98.7% for muscle thickness ≥4 mm.

Four infants (1.1%) underwent a negative surgical exploration; Ultrasound was positive in 3, and negative in 1(who underwent surgery on the basis of a positive upper GI contrast). One US reported as positive had a muscle thickness <4 mm. Two false positive US were performed at peripheral hospitals. One infant had a false positive test feed following a positive ultrasound diagnosis. Two infants had negative test feeds.

**Conclusion:**

A 1% rate of negative exploration in IHPS compares favourably with other studies. However potential causes of error were identified in all 4 cases. Confident diagnosis comprises a combination of positive test feed and an 'in house US' in an alkalotic infant. UGI contrast study should not be used in isolation to diagnose IHPS. If the test feed is negative, strict diagnostic measurements should be observed on US and the pyloric 'tumour' palpated on table under anaesthetic before exploration.

## Background

Before the widespread introduction of US in 1977 [1], the diagnosis of infantile hypertrophic pyloric stenosis (IHPS) was made on the clinical finding of a palpable pyloric mass. An additional suggestive feature in the vomiting child is the characteristic biochemical picture of hypochloraemic hypokalaemic metabolic alkalosis. Although the decreasing incidence of these features (palpable mass and typical biochemical picture) have been attributed to earlier diagnosis and surgical referral [2-4], ultrasound evaluation is often obtained even before attempting a test feed [5] in many infants. The reported diagnostic accuracy of ultrasound in IHPS is variable and operator dependent [6-8]. This study aims to determine the incidence of negative exploration (NL) following a presumptive diagnosis of pyloric stenosis, and to identify factors leading to surgery.

## Methods

The hospital case records of 343 patients who underwent surgical exploration with a presumptive diagnosis of IHPS at the Royal Liverpool Children's Hospital between January 2000 and December 2004 were reviewed. Demographics, clinical findings, preoperative imaging, duration of symptoms, biochemical investigations and operative findings were collected from several sources (case notes, transfer letters, operative reports and computer records). During the period of the study, a defined protocol for diagnosis of IHPS was not used at our centre, and surgical exploration was performed at the discretion of the registrars and consultants based on a variable combination of test feed, US and presence of alkalosis. All recorded test feeds were performed by the duty surgical registrar. A nasogastric tube was used to aspirate the stomach prior to commencing test feeding with the infant in the mother's arms. A test feed was considered positive if it was possible to palpate a pyloric 'olive' during the test while the infant was orally fed with <50 mls of milk or dioralyte. Visible peristalsis in isolation was not criterion for a positive test feed, as it may be present in gastric or intestinal obstruction. The majority (85.5%) of positive ultrasound reports were accompanied by measurements of canal dimensions. At our centre, an US scan was considered to confirm pyloric stenosis if the length of the pyloric canal exceeded 14 mm and the muscle thickness ≥4 mm. Alkalosis was defined as a serum pH ≥7.45 and/or base excess (BE) ≥2.5.

## Results

### Demographics

The mean age at surgery of the study population (296 male and 47 female) was 35 days (range 3–150) and the mean duration of symptoms was 9.5 days (range 1–56).

### Test feed

A test feed was documented in 219/343 (64%) of infants. Of these, 205 (93.6%) were positive. Eighty (38.5%) children underwent pyloromyotomy without US, purely on the basis of a positive test feed.

### Radiology

An US scan was done in 270/343 (79%) children. All but one of the 270 US scans were reported to be positive. Sixty four of these were done in another referring hospital prior to transfer. In 39 studies (including 13 performed at our hospital), the scan was reported as showing pyloric stenosis, without stating the diagnostic measurements. Among the US scans reported as positive, 230 infants had a pyloric canal length more than 14 mm, and 220 had muscle thickness of at least 4 mm. The positive predictive value for an ultrasound (US) diagnosis was 99.1% for canal length ≥14 mm, and 98.7% for muscle thickness ≥4 mm. One hundred and twenty four infants had an US diagnosis without a test feed, and 14 had a negative test feed but positive US diagnosis.

### Biochemical analysis

The case notes and transfer letters from peripheral hospitals were reviewed for evidence of biochemical abnormalities consistent with a diagnosis of pyloric stenosis. One hundred and seventy five (55%) patients had obvious evidence of alkalosis (defined as pH ≥7.45 or BE ≥2.5). Hypokalemia (K<4) was observed in 38 infants and hypochloremia (Cl < 95) in 32.

### Misdiagnoses

Four infants were found to have a normal pylorus at laparotomy (NL) (Table 1). All four patients underwent a minimal myotomy of the normal pylorus. A 'minimal myotomy' is division of the muscle layer while ensuring mucosal integrity (confirmed by an air injection via the nasogastric tube). We have previously seen patients with absence of US features of IHPS who have subsequently developed the radiological features within 3–4 days. The observation of this phenomenon of 'IHPS in evolution' provided the rationale for performing minimal myotomy in these children.

**Table 1:** Patients who had a negative laparotomy (NL)

Patient no	Test feed	UGI	Ultrasound			Biochemistry	
			
			Report	Canal length	Muscle thickness	pH	BE
1	***Negative***	-	Positive	20	4	***7.38***	***-0.1***
2	Positive	-	Positive✶	17	4	***7.42***	***-0.4***
3	***Not done***	-	Positive✶	18	***2.1***	7.46	4.5
4	***Negative***	positive	***Negative***	***10***	***2.2***	***7.44***	5.2

UGI – Upper GI contrast study.

Results which do not reach diagnostic criteria in ***bold italics***.

✶ US done in a peripheral hospital

Infant 1 had negative test feed and a positive ultrasound with normal blood gases. Postoperatively, he developed a wound infection which was treated with oral antibiotics. He was shown to have gastro oesophageal reflux (GOR), which responded to treatment.

Infant 2 was felt to have a palpable pyloric mass after a positive ultrasound from a peripheral hospital. His gases were normal. His vomiting subsided post-operatively after a few days of careful feeding.

Infant 3 had an ultrasound reported from the peripheral hospital as positive, but with a muscle thickness of only 2.1 mm. A test feed was not done. She had an alkalotic picture on blood gases. She collapsed with necrotising enterocolitis 3 days after the surgery and died (without a further laparotomy).

Infant 4 had negative results on both test feed and ultrasound. A diagnosis of pyloric stenosis was based on an upper gastrointestinal contrast study performed at another hospital. He was later diagnosed to have GOR which settled with medical treatment.

## Discussion and conclusion

The typical clinical diagnosis of IHPS is based on the finding of a palpable pyloric 'tumour' in an infant aged between 1 and 6 weeks with a history of vomiting and weight loss. "Projectile" vomiting is not discriminating, as this symptom may feature in any vomiting infant and is common in GOR [9]. Traditionally, a positive test feed established the diagnosis [2,9,10]. However, exclusive reliance on the test feed for diagnosis in pyloric stenosis is associated with false positive rates of 3–14% and false negative rates of 18–33% [6,9,11]. The reported decline in the use of the test feed [2,3,7] is supported by our finding that 39% of the infants in our study did not have the test. The reduced reliance on test feed for diagnosis inevitably diminishes clinical acumen for this mode of diagnosis. In our study, one of the patients in the NL group had a false positive test feed, after a positive ultrasound diagnosis from the referring hospital. The pyloric 'tumour' can be difficult to palpate in the early stages. Interestingly, the role of an element of 'suggestibility' has been reported previously, that if the examiner was aware of a positive US before doing a test feed, the success in palpating the pyloric 'tumour' increased to 46%, compared to the 26% when the examiner was unaware of the positive US [11].

Ultrasonography is now a well established diagnostic modality for the diagnosis of pyloric stenosis. Since its first description by Teele and Smith in 1977 [1], the diagnostic criteria for pyloric stenosis on US have been made more stringent [12-14] in an attempt to minimise false positive results and unnecessary surgery. A combination of definite measurements of canal length and muscle thickness is now expected for confident ultrasonographic diagnosis. Reported over-reliance on US [2,3,15-17] by clinicians has also been attributed earlier presentation of infants and the pressure on early diagnosis in the modern cost-conscious health service [3]. Accuracy in measuring the dimensions of the pylorus is critically dependent on the experience of the individual sonographer, who should see sufficient number of cases to maintain expertise [7]. Currently in the UK NHS, paediatric surgery has become mainly restricted to tertiary centres, and hence with this move, the diagnostic capabilities and experts in paediatric surgical conditions are concentrated in tertiary centres. With these provisions, we now prefer an 'in-house' US for diagnosis of IHPS, defined as one performed by a tertiary centre paediatric radiologist who performs these scans routinely. An upper GI contrast study is not reliable in the diagnosis of IHPS, as it can mistake pylorospasm for canal obstruction [18]. In cases of diagnostic uncertainty between IHPS and GOR, a contrast swallow can help to establish diagnosis of GOR. However, it is clinically difficult to justify using a contrast study in cases where there are typical features of IHPS on US.

Less than 60% of infants had a supporting biochemical picture in our series. The presence of alkalosis supports but does not confirm the diagnosis. In our study, 2 NL infants had evidence of alkalosis at presentation.

The early presentation of children with IHPS may give an equivocal clinical, biochemical and radiological picture. In such circumstances, the infant should be observed and re-evaluated in 1–2 days when the lesion may become demonstrable clinically or radiologically [3]. If there is no palpable 'tumour' on a test feed, criteria for surgery should include definite diagnostic measurements by US followed by finding a palpable pyloric mass on examination under anaesthetic (EUA). In the absence of palpable mass on EUA, we now use upper GI endoscopy to demonstrate features of IHPS and confirm the diagnosis [19,20] before proceeding with pyloromyotomy.

Our 1% rate of NL in IHPS compares favourably with other studies. However, with the benefit of hindsight, in the 4 patients who had negative explorations, the decision to operate may have changed if we strictly adhered to the criteria of positive test feed and an 'in-house' US with definite diagnostic measurements. If these diagnostic criteria are not met, repeat investigations should be considered in order to avoid negative surgical exploration.

## Abbreviations

EUA: examination under anaesthetic; GI: gastrointestinal; GOR: gastro oesophageal reflux; IHPS: infantile hypertrophic pyloric stenosis; NHS: national health service; NL: negative laparotomy; US: ultrasound scan

## Competing interests

The authors declare that they have no competing interests.

## Authors' contributions

DM, SM and RS collected and analysed the data and drafted the manuscript. CB conceived of the study, and participated in its design and coordination. DM, RC, SK and DP participated in the design of the study. CB, PL and SK helped with revisions on the manuscript. All authors read and approved the final manuscript.

## Pre-publication history

The pre-publication history for this paper can be accessed here:


